# Radixin Enhances Colon Cancer Cell Invasion by Increasing MMP-7 Production via Rac1-ERK Pathway

**DOI:** 10.1155/2014/340271

**Published:** 2014-07-21

**Authors:** Qi-Hong Jiang, Ai-Xiang Wang, Yan Chen

**Affiliations:** Department of Gastroenterology, Pu'ai Hospital, Tongji Medical College, Huazhong University of Science and Technology, 50 Gu Tian San Road, Wuhan 430034, China

## Abstract

As a member of the ezrin-radixin-moesin (ERM) family, radixin is overexpressed in many tumor tissues. However, little is known about its role in the progression of colon cancer. So we here aimed to determine the function of radixin in colon cancer cell invasion. Interestingly, we found that the expression of radixin was significantly elevated in colon cancer cells. Knockdown of radixin suppressed the invasion and migration of colon cancer cells. Further, knockdown of radixin inhibited the activation of Rac1 and ERK1/2, and decreased the expression and secretion of MMP-7. In addition, Rac1-ERK signaling pathway was required for the radixin-promoted invasion and MMP-7 production. Together, our findings suggest that radixin enhances the invasion and migration of colon cancer cells. Activation of Rac1-ERK pathway and consequent upregulation of MMP-7 production may contribute to the function of radixin in the regulation of colon cancer cell invasion. Thus, radixin may act as a novel target for the diagnosis and treatment of colon cancer.

## 1. Introduction

Colon cancer is one of the most common malignancies, with high incidence and mortality rate, worldwide [1]. Early diagnostic and therapeutic strategies can improve the overall survival of patients with colon cancer. However, for patients diagnosed with advanced stage disease, colon cancer has often metastasized. Invasion and metastasis still account for the main cause of death in colon cancer. Invasion and metastasis are complex processes that require the participation of multiple pathways and genes [2]. Therefore, it is clear that exploration of the key players that are involved in the invasion and metastasis processes may provide diagnostic and therapeutic targets for colon cancer.

The ezrin/radixin/moesin (ERM) family is composed of ezrin, radixin, and moesin. These proteins act as linkers between the actin cytoskeleton and plasma membrane proteins and play important roles in cell adhesion, polarity, and migration [3]. The ERM proteins belong to the widely distributed membrane-associated band 4.1 superfamily [4]. Structurally, the three ERM proteins share 70–85% amino acid identity and all consist of three domains: a conserved N-terminal domain, *α*-helical region, and a C-terminal domain. The N-terminal domain is called the FERM (4.1-ezrine-radixine-moesin) domain and can interact directly with membrane receptors such as CD44 and CD43. The *α*-helical domain links the N-terminal domain and the C-terminal domain. The C-terminal domain can mediate the link between the actin cytoskeleton and the plasma membrane through F-actin binding site [5]. Increased evidences have shown that ERM proteins are involved in the regulation of tumor progression and metastasis. It is reported that ezrin expression is correlated with metastasis and poor prognosis of many cancers such as nonsmall cell lung cancer, breast cancer, and gastric adenocarcinoma [6–8]. Moesin acts as a potential epithelial-mesenchymal transition (EMT) marker in breast and pancreatic cancer, and the expression level of moesin is linked to tumor size, invasion, and differentiation of oral squamous cell carcinoma [9]. The expression level of radixin is found to be significantly increased in colon tumor tissues [10]. However, little is known with regard to the role of radixin in colon cancer.

In this study, we examined the expression of radixin in colon cancer cells. Further, we investigated the role of radixin in colon cancer cell invasion and migration and elucidated the possible molecular mechanism of radixin in regulating cell invasion. Finally, we identified that radixin promoted invasion and migration of colon cancer cells by activating Rac1-ERK pathway and by increasing MMP-7 production.

## 2. Materials and Methods

### 2.1. Cell Culture and Reagents

All cell lines were obtained from American Type Culture Collection (Manassas, VA, USA). The nonmalignant human colonic epithelial cell line NCM460 was cultured in M3D media supplemented with 10% fetal bovine serum (FBS). The human colon cancer cell lines HT-29, Caco-2, HCT116, and LoVo were grown in DMEM with 10% FBS. Antibodies against radixin, ERK1/2, and *β*-actin were purchased from Santa Cruz (Camarillo, CA, USA). Antibodies against Rac1 and phospho-ERK1/2 were purchased from Cell Signaling Technology (Danvers, MA, USA). Rac1 specific inhibitor NSC23766 and ERK1/2 specific inhibitor U0126 were obtained from Sigma (St Louis, MO, USA).

### 2.2. Real-Time PCR

Total RNA of the cultured cells was isolated by Trizol reagent (Invitrogen, Carlsbad, CA, USA). To obtain cDNA, 2 *μ*g of RNA was subjected to reverse transcription with Quantscript cDNA kit (TIANGEN, Shanghai, China). Real-time PCR was performed with cDNA, real-time PCR Master Mix, and primers. The following primers of radixin (forward primer: 5′-TATGCTGTCCAAGCCAAGTATG-3′, reverse primer: 5′-CGCTGGGGTAGGAGTCTATCA-3′), MMP-7 (forward primer: 5′-GAGTGAGCTACAGTGGGAACA-3′, reverse primer: 5′-CTATGACGCGGGAGTTTAACAT-3′), and *β*-actin (forward primer: 5′-ATAGCACAGCCTGGATAGCAACGTAC-3′, reverse primer: 5′-CACCTTCTACAATGAGCTGCGTGTG-3′) were used. The real-time PCR cycle parameters were 10 min at 95°C and 40 cycles of 15 s at 95°C and 1 min at 60°C. Gene expression was normalized against the expression of *β*-actin gene and quantified by the 2^−ΔΔCt^ method.

### 2.3. Western Blot Analysis

After washing with sterile PBS, cells were lysed in RIPA lysis buffer supplemented with protease and phosphatase inhibitors (Applygen Technologies Inc., Beijing, China). The protein concentrations were determined by the BCA method. Then 50 *μ*g of protein was loaded in SDS-PAGE gel and electrophoresed under denaturing and reducing conditions. The resolved proteins in the gel were transferred to PVDF membranes. The membranes were further immunoblotted with primary antibodies overnight at 4°C. Next, the membranes were immunoblotted with secondary antibodies for 1 h at room temperature. Then the bands were visualized using electrochemiluminescence (ECL) reagents (Applygen Technologies Inc.) and Bio-Mark MS films.

### 2.4. siRNA Transfection

The control siRNA (5′-UGGUUUACAUGUUUUCUGA-3′) and radixin siRNA (5′-AUGUUCUUCAUGCCAGUUC-3′) were obtained from Genepharma (Shanghai, China). HCT116 and LoVo cells were transfected with siRNAs by using Lipofectamine RNAi Max (Invitrogen, Carlsbad, CA, USA). After observing the knockdown efficiency by western blotting 48 h later, the transfected cells were used in the subsequent experiments.

### 2.5. Invasion and Migration Assays

The upper chambers of 24-well Transwell plate (Costar, San Diego, CA, USA) were coated with (for invasion assay) or without (for migration assay) Matrigel (BD, Franklin Lakes, NJ, USA) before the experiments were carried out. The cells were adjusted at 2 × 10^5^/mL in each group. The upper chambers were loaded with 200 *μ*L of cell suspension, while the lower chambers were loaded with 500 *μ*L of DMEM containing 30% FBS. After incubation in a CO_2_ incubator at 37°C for 12 h, the invaded or migrated cells were fixed and stained with crystal violet. The number of cells was counted from five randomly visual fields. Data were expressed as the percentage of the control.

### 2.6. Rac1 Activity Assay

Cells transfected with control siRNA or radixin siRNA were lysed in RIPA lysis buffer and then subjected to Rac1 activity assay kit (Millipore, MA, USA), according to manufacturer's protocols. Briefly, the lysates were incubated with p21 activated kinase-GST fusion protein and Glutathione-Sepharose beads at 4°C for 1 h. Then the beads were washed and the proteins were collected. Total and GTP bound Rac1 were determined by immunoblotting against Rac1.

### 2.7. ELISA Assay

Cell supernatant was collected from each group and then was centrifuged for 10 min at 10 000 rpm. MMP-7 ELISA kit was purchased from Sigma and used to examine the MMP-7 secretion in colon cancer cells, according to manufacturer's protocols. Cells were lysed in RIPA lysis buffer and total protein concentrations were calculated. MMP-7 concentration was determined by normalizing to total protein concentrations.

### 2.8. Statistical Analysis

All experiments were performed in triplicates. All data represented mean ± SD. Statistical analysis was performed with SPSS 17.0 software. Student's *t*-test was used to analyze the significance between two groups and one-way analysis of variance (ANOVA) was used to analyze the significance between multiple groups. Statistical significance was set at *P* < 0.05.

## 3. Results

### 3.1. Radixin Expression Is Significantly Elevated in Colon Cancer Cells

**Figure 1:**
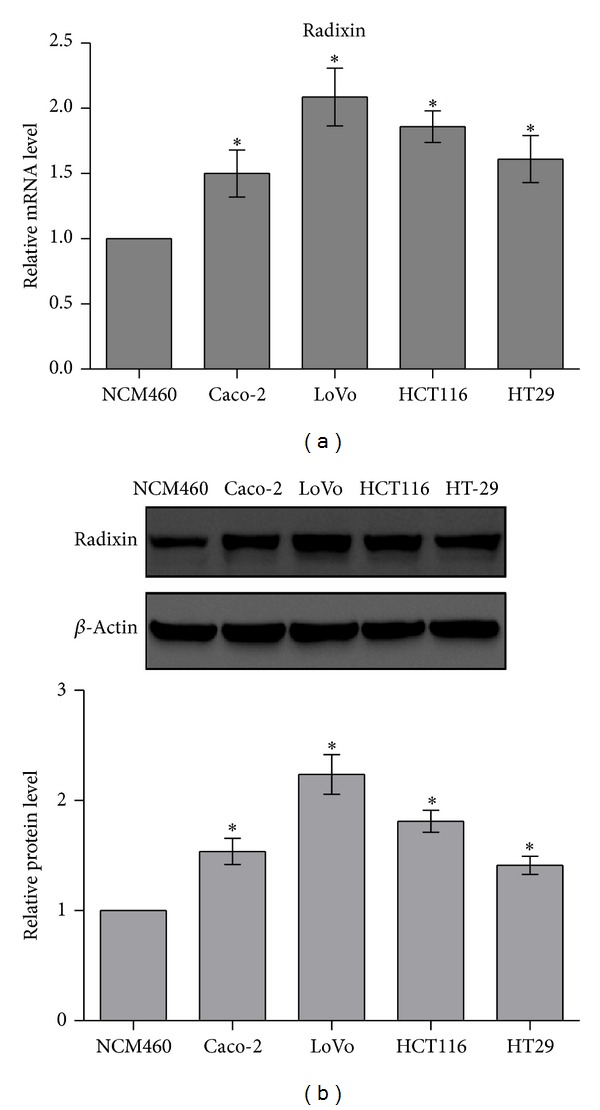
Radixin expression was upregulated in colon cancer cells. (a) Radixin mRNA level in NCM460, HT-29, Caco-2, HCT116, and LoVo cells was determined by real-time PCR. (b) Radixin protein level in NCM460, HT-29, Caco-2, HCT116, and LoVo cells was determined by western blotting. *P < 0.05.

Using real-time PCR and western blotting, we detected the expression of radixin in nonmalignant human colonic epithelial NCM460 cells and colon cancer HT-29, Caco-2, HCT116, and LoVo cells. Interestingly, we found that compared with NCM460 cells, the expression of radixin was markedly increased in all four colon cancer cells (Figures 1(a) and 1(b)), indicating that radixin may act as an important player in colon cancer.

### 3.2. The Expression of Radixin in Colon Cancer Cells Was Silenced by siRNA

**Figure 2:**
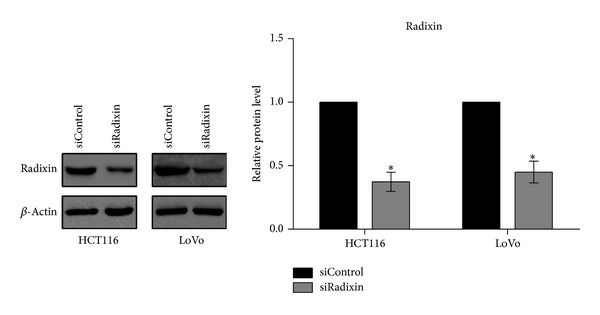
Knockdown efficiency of radixin was examined by western blot analysis. HCT116 and LoVo cells were transfected with either control siRNA (siControl) or radixin siRNA (siRadixin) for 48 h. The knockdown efficiency was examined by western blot analysis. *P < 0.05.

To investigate the role of radixin in colon cancer cell, a radixin-specific siRNA was introduced to silence radixin expression in HCT-116 and LoVo cells. The knockdown efficiency was up to 70% as assessed by western blotting, confirming that this radixin siRNA can prominently attenuate the expression of radixin in HCT-116 and LoVo cells (Figure 2).

### 3.3. Radixin Promotes the Invasion and Migration of Colon Cancer Cells

**Figure 3:**
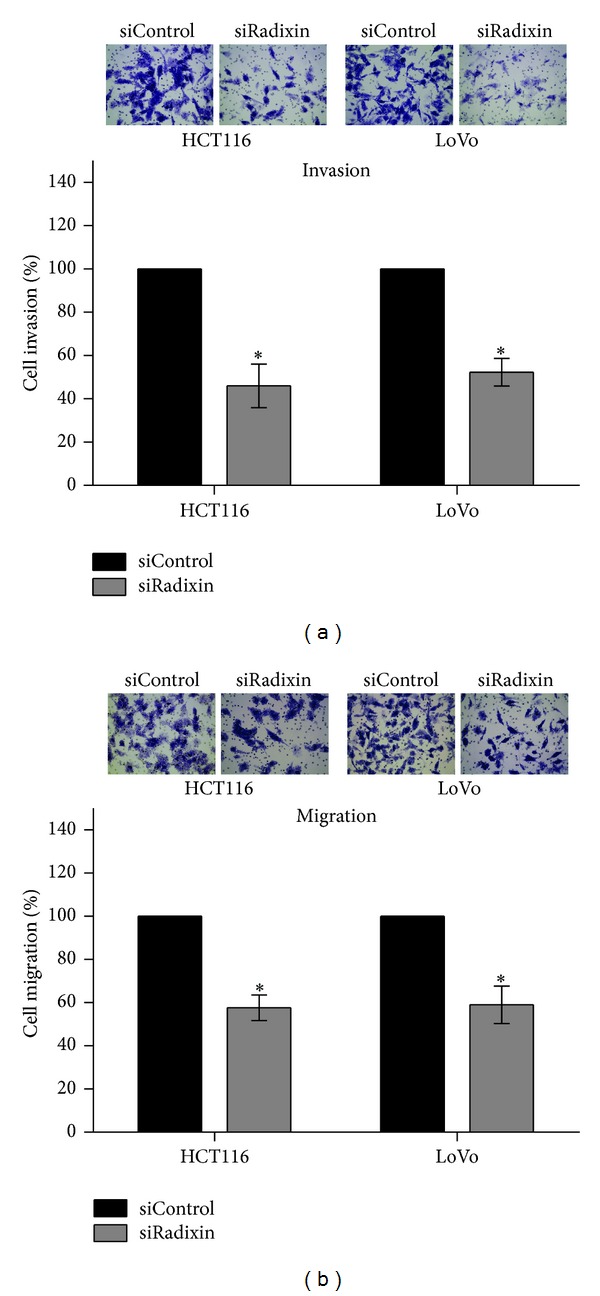
Knockdown of radixin by siRNA suppressed the invasion and migration of colon cancer cells. After transfection with radixin siRNA or control siRNA, cells were subjected to invasion and migration assays. (a) Effect of radixin knockdown on the invasion of HCT116 and LoVo cells. (b) Effect of radixin knockdown on the migration of HCT116 and LoVo cells. *P < 0.05.

To examine the function of radixin in colon cancer cell invasion and migration, invasion and migration assays were performed with control siRNA cells (siControl) and radixin siRNA cells (siRadixin). The results showed that the invasion and migration abilities of radixin siRNA cells were significantly suppressed compared with control siRNA cells, suggesting that radixin can enhance the invasion and migration of colon cancer cells (Figures 3(a) and 3(b)).

### 3.4. Rac1 Activated by Radixin Is Involved in the Invasion and Migration of Colon Cancer Cells

**Figure 4:**
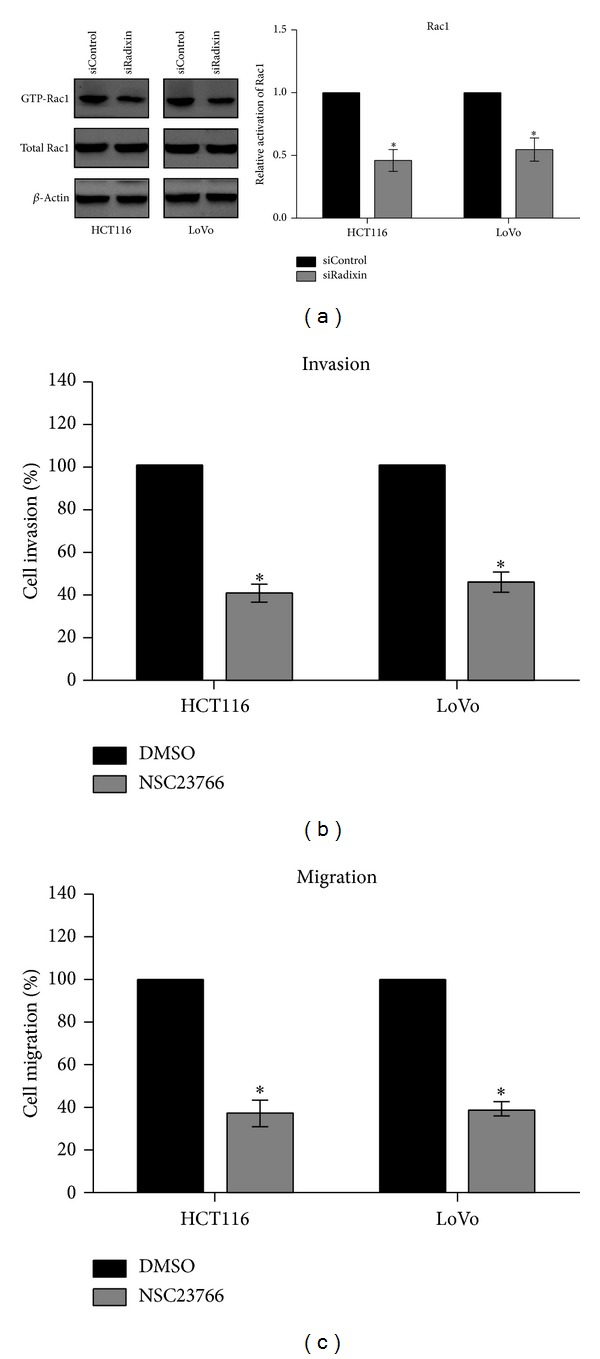
Radixin enhanced the invasion and migration of HCT116 and LoVo cells through Rac1 activation. (a) Cells were transfected with either control siRNA or radixin siRNA for 48 h. Then the amount of GTP binding protein Rac1 was analyzed by Rac1 activity assay kit. (b) and (c) Cells were pretreated with NSC23766 or DMSO for 1 h and then were subjected to invasion and migration assays. *P < 0.05.

Rho GTPase Rac1 is closely related with cell invasion and metastasis of cancers [11]. Here, we found that knockdown of radixin led to a significant decrease in Rac1 activation, suggesting the involvement of radixin in the regulation of Rac1 activation (Figure 4(a)). To investigate the role of Rac1 activation in the radixin-promoted invasion and migration, we pretreated HCT116 and LoVo cells with 100 *μ*M NSC23766 (Rac1 inhibitor) for 1 h. Then we subjected the treated or untreated cells to invasion and migration assays. The results showed that inactivation of Rac1 suppressed the invasion and migration of both HCT116 and LoVo cells (Figures 4(b) and 4(c)), suggesting that Rac1 is required for the radixin-promoted invasion and migration.

### 3.5. Rac1-ERK Pathway Contributes to the Radixin-Promoted Invasion and Migration

**Figure 5:**
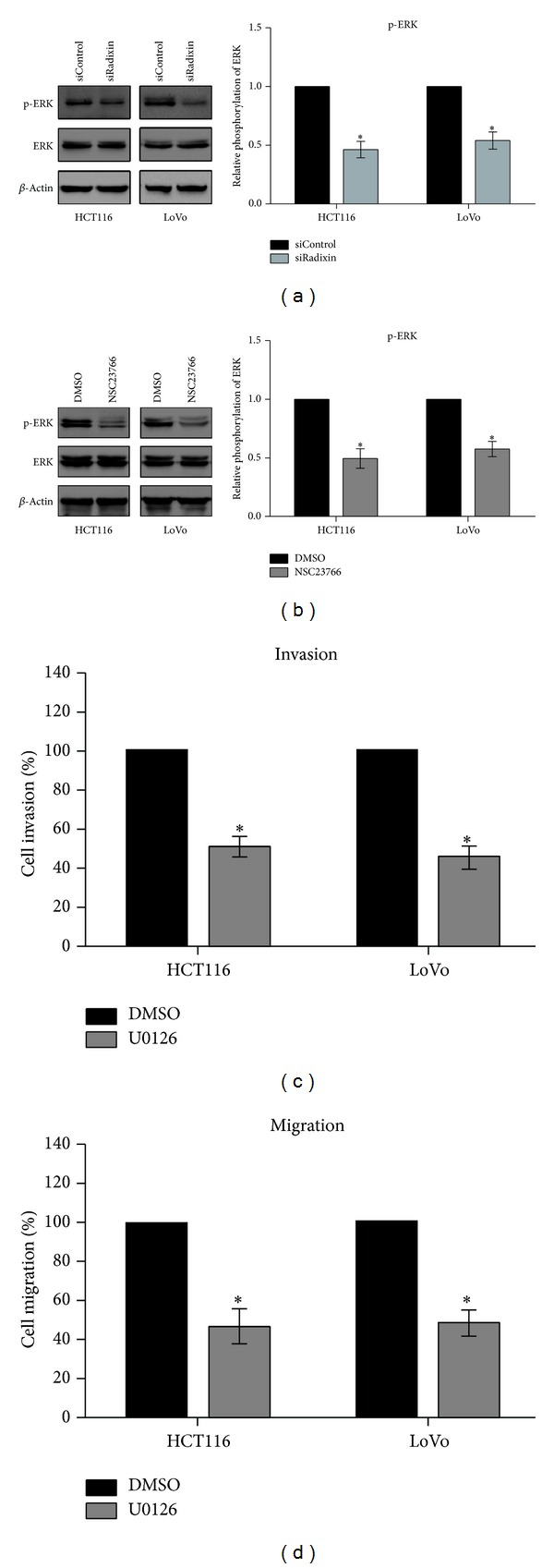
Rac1-ERK pathway was involved in the radixin-mediated invasion and migration of colon cancer cells. (a) After transfected with either control siRNA or radixin siRNA for 48 h, the phosphorylation of ERK1/2 was detected by western blotting. (b) After incubation with NSC23766 or DMSO for 1 h, the phosphorylation of ERK1/2 was detected by western blotting. (c) and (d) Cells were pretreated with U0126 or DMSO for 30 min and then were subjected to invasion and migration assays. *P < 0.05.

Next, using western blotting, we found that knockdown of radixin inhibited the activation of ERK1/2 in both HCT-116 and LoVo cells (Figure 5(a)). Furthermore, to analyze the effect of Rac1 on the radixin-regulated ERK1/2 activation, we pretreated HCT-116 and LoVo cells with 100 *μ*M NSC23766 (Rac1 inhibitor) for 1 h and then observed the phosphorylation of ERK1/2. Using western blotting, we found that inhibition of Rac1 attenuated the phosphorylation of ERK1/2 (Figure 5(b)). These results suggest that radixin induces ERK1/2 activation via regulation of Rac1 activity. To determine the function of ERK pathway in colon cancer cell invasion and migration, cells were subjected to invasion and migration assays after treatment with 10 *μ*M U0126 (ERK1/2 inhibitor) for 30 min. Our results demonstrated that U0126 treatment reduced the invasion and migration of colon cancer cells (Figures 5(c) and 5(d)). Together with the results shown in Figure 4, these data indicate that radixin enhances colon cancer cell invasion and migration by activating Rac1-ERK pathway.

### 3.6. Radixin Upregulates MMP-7 Production via Rac1 and ERK1/2 Activation

**Figure 6:**
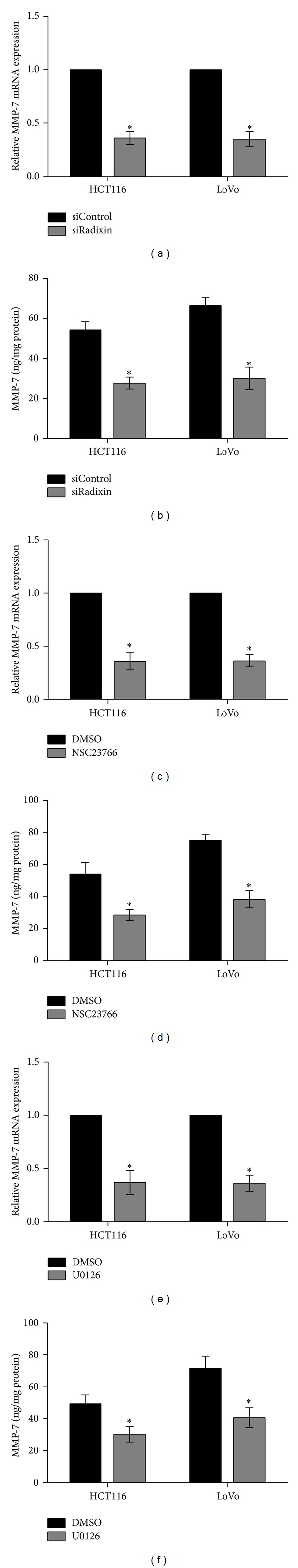
Rac1-ERK pathway was required for the radixin-mediated MMP-7 production. (a) and (b) After knockdown of radixin by siRNA, the production of MMP-7 was observed by real-time PCR and ELISA assay. (c) and (d) After incubation with NSC23766 or DMSO for 12 h, MMP-7 production was determined by real-time PCR and ELISA assay. (e) and (f) After incubation with U0126 or DMSO for 12 h, MMP-7 production was detected by real-time PCR and ELISA assay. *P < 0.05.

MMP-7 is crucial for the invasion and metastasis of colon cancer [12]. Here, using real-time PCR and ELISA assay, we found that compared with control siRNA cells the expression and secretion of MMP-7 were greatly suppressed in radixin siRNA cells (Figures 6(a) and 6(b)). Next, we incubated colon cancer cells with NSC23766 or U0126 for 12 h, respectively. Then we examined the expression and secretion of MMP-7 by real-time PCR and ELISA assay. We found that blocking of Rac1 and ERK1/2 activation both downregulated MMP-7 expression and secretion (Figures 6(c)–6(f)). Since radixin can induce Rac1 and ERK1/2 activation as described above, these results suggest that radixin influences MMP-7 production via Rac1 and ERK1/2 activation in colon cancer cells.

## 4. Discussion

**Figure 7:**
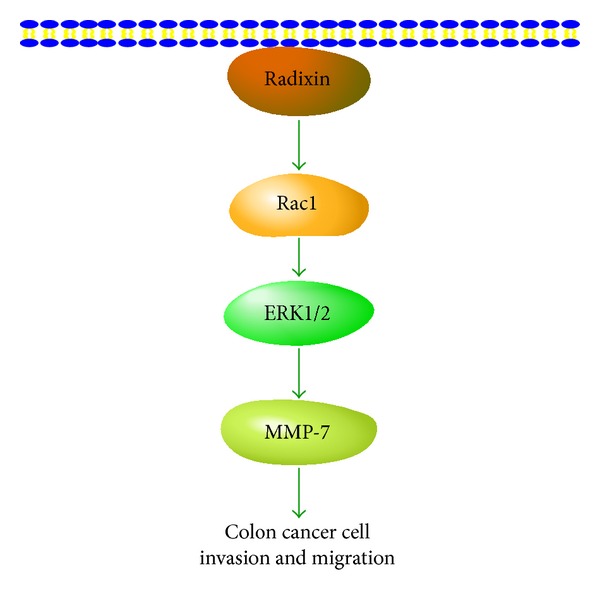
Diagram depicting the possible molecular mechanism of radixin in colon cancer cell invasion and migration.

Among the ERM proteins, radixin has been known as an important player in cell-cell and cell-matrix contacts, as well as in the reorganization of actin cytoskeleton [13]. In the present study, we found that the expression of radixin was markedly elevated in colon cancer cells. Further findings showed that radixin induced the activation of Rac1-ERK pathway, leading to an increase in MMP-7 production, thereby contributing to the invasion and migration of colon cancer cells (Figure 7). To our knowledge, this study is the first to reveal the molecular mechanism of radixin in colon cancer cell invasion.

Clinical studies have observed the expression pattern of radixin in different cancers. In breast cancer and lung cancer, reduced expression of radixin has been found in tumor tissues compared with the adjacent benign tissues [14, 15]. On the contrary, the expression of radixin is greatly increased in prostate cancer, colon cancer, pancreatic cancer, and gliomas, and elevated level of radixin is closely related to the poor prognosis of cancers [10, 16, 17]. Our results showed that the expression of radixin was increased in colon cancer cells, further confirming a role of radixin in colon cancer. Experimental studies have revealed that radixin contributes to the invasion and metastasis of gastric cancer cells and glioma cells [18, 19], and silencing of radixin inhibited the invasion and growth of pancreatic cancer cells* in vitro* and* in vivo* [20]. However, little is known about the function of radixin in colon cancer. Here, using invasion assay and migration assay, we found that knockdown of radixin by siRNA suppressed the invasion and migration of colon cancer cells, implicating that radixin may be a key mediator in the regulation of colon cancer cell invasion.

Rho GTPases are a family of GTP binding proteins that mediate actin dynamics and act as important modulators in various steps of cancer progression [21]. As one member of Rho GTPases, Rac1 activation can affect the actin cytoskeleton and mediate the formation of lamellipodia and filopodia [22]. In addition, Rac1 activation attenuates cell-cell adhesion by downregulation of E-cadherin [23] and increases the expression and secretion of metalloproteinases (MMPs) [24]. Thus, activation of Rac1 contributes to the migration, invasion, and metastasis of cancers [11]. It is reported that radixin can regulate cell migration and cell-cell adhesion through Rac1 activation in prostate cancer cells [25]. Consistently, we found that radixin was necessary for the activation of Rac1 in colon cancer cells. Selective blockade of Rac1 activity suppressed the invasion and migration of HCT116 and LoVo cells, implying the involvement of Rac1 in the radixin-mediated invasion and migration of colon cancer cells.

ERK pathway has a pivotal role in the regulation of cancer development, such as cell proliferation, invasion, and metastasis [26]. Of note, ezrin as well as moesin can mediate the activation of ERK pathway in cancer cells [27, 28]. However, the relationship between radixin and ERK pathway remains unclear. Here, our findings showed that radixin was able to induce ERK1/2 activation. Studies have reported that Rac1 can regulate multiple intracellular signaling pathways in colon cancer, such as PI3K/AKT and MAPK pathways [29]. Our further experiments found that Rac1 was required for the radixin-mediated activation of ERK1/2. Moreover, we found that ERK pathway contributed to the invasion and migration of colon cancer cells. Since radixin can regulate the activation of Rac1 and ERK1/2 and Rac1-ERK signaling is essential for the colon cancer cell invasion and migration, it is possible that radixin promotes the invasion and migration through activation of Rac1-ERK pathway.

Belonging to the MMP family, MMP-7 is overexpressed in many cancers including colon cancer and is associated with the metastasis and progression of cancers [30, 31]. Here, our results proved that radixin increased the expression and secretion of MMP-7. Rac1 and ERK1/2 activation have been found to be a respond to the increased expression of MMPs in most cancer cells [32]. In this study, we found that blocking of Rac1-ERK signaling decreased the expression and secretion of MMP-7. As radixin can regulate the activation of Rac1 and ERK1/2, these data strongly support the notion that radixin mediates MMP-7 production via activation of Rac1 and ERK1/2.

In summary, our present work demonstrates that radixin can promote the invasion of colon cancer cells by activating Rac1-ERK pathway and upregulating MMP-7 production. Therefore, radixin may act as a promising therapeutic target for the treatment of colon cancer.
